# Chronic murine toxoplasmosis is defined by subtle changes in neuronal connectivity

**DOI:** 10.1242/dmm.014183

**Published:** 2014-02-13

**Authors:** Alexandru Parlog, Laura-Adela Harsan, Marta Zagrebelsky, Marianna Weller, Dominik von Elverfeldt, Christian Mawrin, Martin Korte, Ildiko Rita Dunay

**Affiliations:** 1 Institute of Medical Microbiology, Otto-von-Guericke University, 39120-Magdeburg, Germany.; 2 Department of Radiology, Medical Physics, University Medical Center Freiburg, 79106-Freiburg, Germany.; 3 Zoological Institute, Division of Cellular Neurobiology, University of Braunschweig, 38106-Braunschweig, Germany.; 4 Department of Neuropathology, Otto-von-Guericke University, 39120-Magdeburg, Germany.; 5 Helmholtz Centre for Infection Research, AG NIND, 38106-Braunschweig, Germany.

**Keywords:** Parasites, Behavioral manipulation, Neuronal connectivity

## Abstract

Recent studies correlate chronic *Toxoplasma gondii* (*T. gondii*) infection with behavioral changes in rodents; additionally, seropositivity in humans is reported to be associated with behavioral and neuropsychiatric diseases. In this study we investigated whether the described behavioral changes in a murine model of chronic toxoplasmosis are associated with changes in synaptic plasticity and brain neuronal circuitry. In mice chronically infected with *T. gondii*, magnetic resonance imaging (MRI) data analysis displayed the presence of heterogeneous lesions scattered throughout all brain areas. However, a higher density of lesions was observed within specific regions such as the somatosensory cortex (SSC). Further histopathological examination of these brain areas indicated the presence of activated resident glia and recruited immune cells accompanied by limited alterations of neuronal viability. *In vivo* diffusion-tensor MRI analysis of neuronal fiber density within the infected regions revealed connectivity abnormalities in the SSC. Altered fiber density was confirmed by morphological analysis of individual, pyramidal and granule neurons, showing a reduction in dendritic arbor and spine density within the SSC, as well as in the hippocampus. Evaluation of synapse efficacy revealed diminished levels of two key synaptic proteins, PSD95 and synaptophysin, within the same brain areas, indicating deficits in functionality of the synaptic neurotransmission in infected mice. Our results demonstrate that persistent *T. gondii* infection in a murine model results in synaptic deficits within brain structures leading to disturbances in the morphology of noninfected neurons and modified brain connectivity, suggesting a potential explanation for the behavioral and neuropsychiatric alterations.

## INTRODUCTION

*Toxoplasma gondii* (*T. gondii*) is a widespread intracellular parasite that is able to infect all warm-blooded species. The parasite has a complex life cycle in various hosts (Montoya and Liesenfeld, 2004; Randall and Hunter, 2011; Hunter and Sibley, 2012). Human infections occur mainly through oral ingestion of contaminated food or water. Approximately 30% of the entire human population is estimated to be infected with *T. gondii* without major clinical manifestations in immunocompetent individuals (Montoya and Liesenfeld, 2004; Munoz et al., 2011). After dissemination throughout the body, the parasites reach immune-privileged areas such as the central nervous system (CNS) (Ferguson et al., 1991; Dubey, 2009). Latent toxoplasmosis is characterized by bradyzoite-containing cyst development within neurons, where the parasites stay hidden from the immune system (Lyons et al., 2002; Dubey, 2009).

Independent studies carried out in recent years emphasized the ability of *T. gondii* infection to contribute to neurological and psychiatric disorders. Clinically, chronic *T. gondii* infection is often associated with symptoms ranging from slight personality changes and altered psychomotor performance (Flegr et al., 2002; Yereli et al., 2006; Stock et al., 2013; Beste et al., 2014) to more severe ones, such as schizophrenia-spectrum disorders, self-directed violence, mood disorders and psychosis (Arling et al., 2009; Zhu, 2009; Hinze-Selch et al., 2010; Pedersen et al., 2012; Fabiani et al., 2013). Several studies based on *T. gondii* seropositivity emphasized that individuals with schizophrenia have an increased incidence of *T. gondii* infection compared with control volunteers (Torrey and Yolken, 2003; Brown, 2006; Wang et al., 2006; Hinze-Selch et al., 2007). However, a clear link between persistent *T. gondii* infection and neurological disorders could not be discerned.

A number of studies have demonstrated the ability of *T. gondii* to manipulate the behavior of rodents in relation to predator-prey interactions (Hutchinson et al., 1980; Webster et al., 1994; Berdoy et al., 1995; Berdoy et al., 2000). The infection not only reduced the natural aversion of rats and mice to cat odor, but instead attracted them (Berdoy et al., 2000; Vyas et al., 2007; Haroon et al., 2012). Furthermore, this behavioral adaptation was reported to be highly specific and not due to destruction of the olfactory regions of the brain (Lamberton et al., 2008). Another recent study, which consequently used an identical murine model of toxoplasmosis as implemented in our study, described motor coordination and sensory deficits, whereas cognitive functions were not altered (Gulinello et al., 2010). However, the underlying neurobiological mechanisms by which *T. gondii* alters brain functions remain largely unclear. Collectively, all previous results point toward abnormal modulation of neuroconnectivity induced by direct or indirect parasite-host interaction in specific brain areas, which could explain the reported behavioral alterations. A compelling number of neuroimaging studies on individuals with schizophrenia, bipolar disorders, psychosis, depression and obsessive compulsive disorders have provided evidence of white matter abnormalities, synaptic plasticity deficits and aberrant brain connectivity or dysconnectivity, leading to abnormal functional integration of brain processes (Lawrie et al., 2003; Goghari et al., 2005; Menzies et al., 2008; Vasic et al., 2009; Zalesky et al., 2011; Woodward et al., 2012; Anticevic et al., 2013). Interestingly, a voxel-based morphometry magnetic resonance imaging (MRI) study on *T. gondii*-positive schizophrenic patients reported a significant reduction of gray matter volume in cortical regions, indicating changes in the neuropil. The anatomic substrate of the gray matter volume reduction was not investigated, representing a significant omission (Horacek et al., 2012). The hallmark of persistent *T. gondii* infection is permanent resident glial-cell activation in the CNS (Strack et al., 2002; Suzuki, 2002a; Wang and Suzuki, 2007). Additionally, immune cells from the periphery migrate into the brain and contribute to a perpetual production of inflammatory cytokines and antiparasitic active molecules (Suzuki, 2002b; Hunter and Sibley, 2012). This inflammatory milieu as well as the activated immune cells can distinctively interact with the neurons, modifying their functions and morphology (Coogan and O’Connor, 1997; Curran and O’Connor, 2001; Spedding and Gressens, 2008; Ousman and Kubes, 2012; Kettenmann et al., 2013).

**TRANSLATIONAL IMPACT**

**Clinical issue**

*Toxoplasma gondii* (*T. gondii*) is an obligate intracellular parasite that is widely distributed throughout all warm-blooded animals and humans, with a high tropism for the central nervous system (CNS). Upon transmission to the host, the parasite can establish a lifelong infection characterized by the development of bradyzoite-containing cysts within neurons, where the parasite hides from the host’s immune system. Recently, chronic toxoplasmosis has been correlated with specific behavioral and neurological alterations in both rodents and humans, but the neurobiological mechanisms by which *T. gondii* alters brain functions remain unclear. Specifically, although the functional deficits of individual infected neurons have been demonstrated, the consequences of these deficits on the brain’s wiring scheme are largely unknown.

**Results**

Here, the authors investigate the pathological changes in different brain regions induced by chronic toxoplasmosis in mice. *In vivo* analysis of neuronal fiber density and fiber continuity in the infected regions, using diffusion-tensor MRI and a fiber-tracking methodology, reveals impaired local connectivity, particularly within the somatosensory areas. These observations were paralleled by reduced expression of two cytoskeletal proteins in the somatosensory cortex and hippocampus. Furthermore, detailed morphological analyses of individual, non-infected, neurons from these brain areas reveal decreased dendritic complexity and dendritic spine disorganization in infected mice compared with control mice. Finally, in line with these morphological alterations, the authors report modifications of the expression level of specific proteins that regulate key synaptic functions in the same brain areas.

**Implications and future directions**

These findings indicate that, upon latent infection with *T. gondii*, marked neuroanatomical changes occur in CNS regions that are relevant for normal behavior, and establish a murine model for translational studies of chronic toxoplasmosis. Importantly, the neuroanatomical changes reported here are similar to those previously reported in individuals with neuropsychiatric diseases such as schizophrenia-like disorders, which have been associated with *T. gondii* infection in some studies. The pathological alterations uncovered in this work might therefore represent a starting point for the elucidation of the circumstances and the cellular mechanisms responsible for the neurological and behavioral impairments recorded in humans infected with *T. gondii*. Finally, an in-depth understanding of the relationship between *T. gondii* infection and neurological disorders in general could lead ultimately to the development of specific protocols for the prevention and treatment of a range of neurological and psychiatric disorders.

In this study we report changes in mouse brain connectivity and synaptic communication with chronic toxoplasmosis, which we believe is crucial to explain the behavioral shift. We have mapped the localization and characterized histologically the cellular composition of the microlesions induced by *T. gondii* infection within the murine brain. *In vivo*, diffusion-tensor MRI (DT-MRI) and fiber tracking methods were used to investigate changes in brain connectivity patterns in the lesion areas. We showed altered fiber density and loss of fiber continuity in infected cortical regions, leading to local and generalized white matter connectional architecture impairments. Importantly, we described that persistent *T. gondii* infection particularly affects cortical neurons, manifesting changes in morphology and dendritic bundling, therefore providing a possible explanation for the defective connectivity described by DT-MRI. Finally, we correlated the observed morphological alterations of the neurons with modifications of the expression level of specific proteins regulating synaptic functions.

## RESULTS

### Distribution of *T. gondii*-induced lesions: MRI vs histopathological investigations

**Fig. 1.**
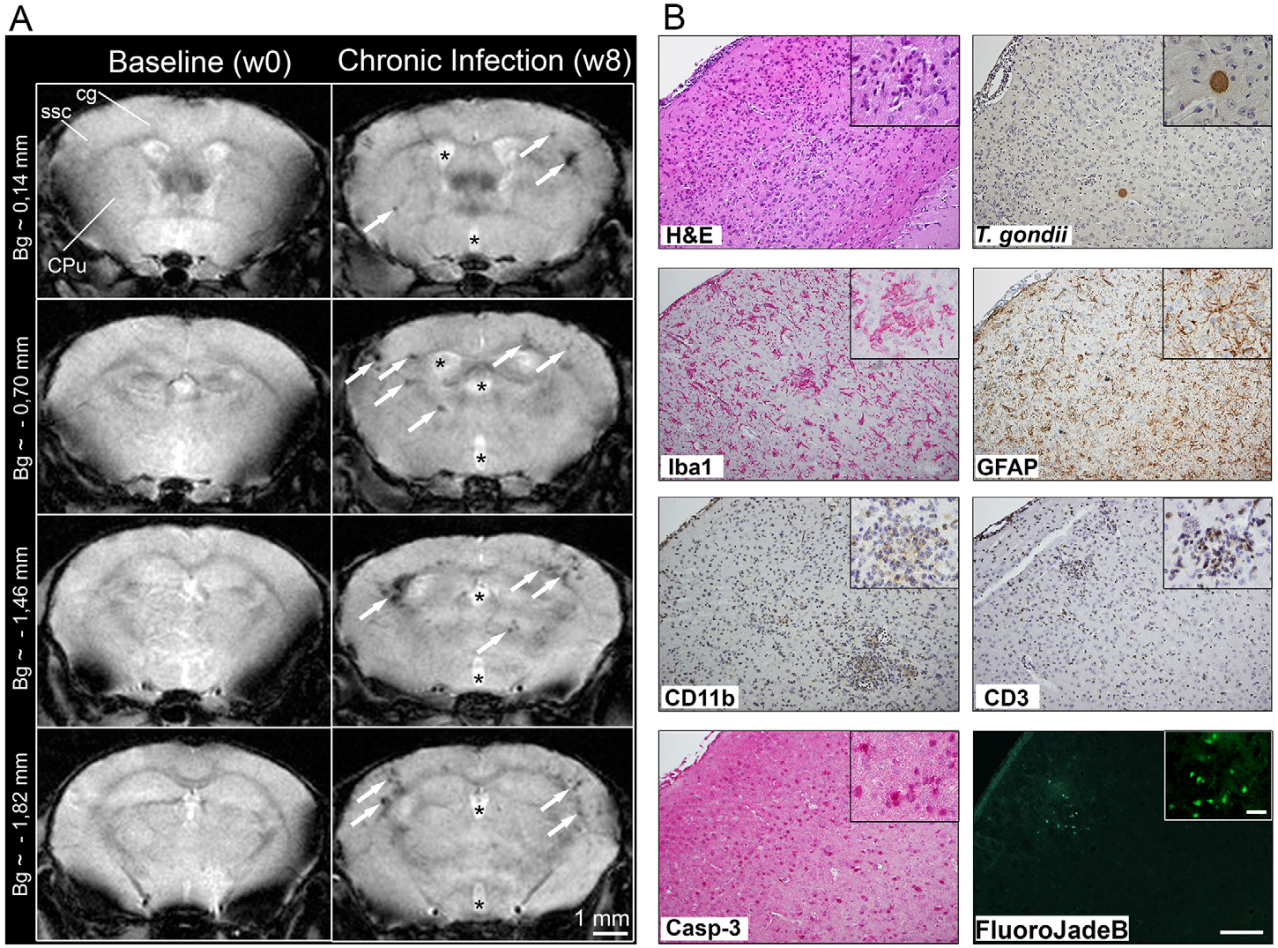
**Pathological changes identified by MRI and histopathological examination of the murine brain chronically infected with *T. gondii*.** (A) Representative T2*-weighted MR images, acquired before infection (baseline) and at 8 weeks post-infection (chronic), depicting *T. gondii*-induced brain microlesions throughout four axial brain slices. Note the changes in the lateral and third ventricle size (asterisks) and the localization of the lesions (arrows) within the cortex but also in the striatum and along the white matter fiber tracks (ssc, somatosensory cortex; cg, cingulum; CPu, caudate putamen; Bg, bregma). (B) In the cortical regions where hypointense T2*-weighted lesions were observed, hematoxylin-eosin and anti-*T. gondii* stainings revealed a higher density of cells, parenchymal micro-hemorrhage and the presence of intact *T. gondii* cysts. Closer examination using anti-Iba-1 and anti-GFAP antibodies indicated diffuse activation of microglia and astrocytes, respectively. This was followed by recruitment of CD11b-positive cells, indicating microglia cells as well as brain-recruited macrophages. Numerous recruited T cells were detected by anti-CD3 antibody. Apoptotic cells, highlighted by an anti-caspase-3 staining, were scattered throughout cortical areas. Fluoro-Jade B staining revealed a low number of degenerating neurons limited to the inflammatory foci (*T. gondii*-infected mice *n*=7; four to six coronal slides per mouse were analyzed). Scale bars in B: 100 μm, 20 μm in insets.

To determine the pattern of brain microstructural alterations induced by chronic *T. gondii* infection in a murine model, *in vivo* noninvasive MRI was performed at high magnetic field (9.4 T). T2*-weighted imaging was used to identify and anatomically localize the preferential lesion sites of parasite-induced brain pathology. Multiple foci of hypointensity were visualized with T2*-based contrast in various cortical and subcortical brain areas (Fig. 1A). A pattern of high density of hypointense lesions was evident in the somatosensory (SSC) and motor cortices, hippocampus and striatal brain structures of all the infected mice. Interestingly, in T2-weighted imaging, such regions appeared generally unaffected, demonstrating the superiority of T2*-weighted imaging for assessing the *T. gondii*-induced brain pathology. The excellent contrast obtained with the T2-weighted imaging allowed generation of group-averaged (control versus *T. gondii*-infected groups) T2-weighted images and the segmentation of the ventricles. Furthermore, a significant ventricle enlargement (increase with 23.5%) could be measured in the *T. gondii* infected brains.

To further define the composition of microstructural alterations leading to the changed T2*-contrast in the infected mice, brains of mice used for MRI studies were analyzed *ex vivo* using various neuropathological markers (Fig. 1B). Scattered inflammatory infiltrates were detected throughout the whole brain, as well as only minor meningeal alterations and edemas being observed (hematoxylin-eosin staining). We focused our analysis on the somatosensory cortex (regions between bregma 0.14 mm and bregma −1.82 mm), where we observed a high density of hypointense lesions on T2*-weighted images of the infected mice. The number of *T. gondii* cysts was randomly distributed with no preference to a specific brain area. In each slide, six to ten parasite cysts were detected within the cortex, one to two cysts within the hippocampus and four to six cysts in the other remaining brain areas. Total cyst number in the whole brain varied from 400 to 600. The cyst wall around the bradyzoites remained intact and extracellular parasites were not present, indicating persistent rather than active *T. gondii* infection (Fig. 1B). Interestingly, clusters of activated immune cells were not exclusively surrounding the cysts. Activated resident microglia cells were distributed ubiquitously in the whole brain with preference to the developing cortical inflammatory nodules (Iba1-positive). Activated astrocytes manifested evenly throughout the brain parenchyma (GFAP-positive). Moreover, perivascular cuffs were constituted mainly by recruited mononuclear cells (CD11b-positive), with few neutrophil granulocytes and lymphocytes (CD3-positive) (Fig. 1B). Cell apoptosis (marked by Casp-3) was observed, indicating general cell death as a consequence of chronic *T. gondii* infection. To clarify whether neurons were affected, we employed a specific staining (Fluoro-Jade B) that only detects degenerating neurons. Fluoro-Jade-B-labeled cells were observed only sporadically in the inflammatory foci within the cortex and hippocampus, indicating that the majority of neurons in those regions did not die (Fig. 1B). Interestingly, we captured a few infiltrating macrophages amid their active phagocytosis process, removing myelin residues around damaged axons (supplementary material Fig. S1).

### Chronic *T. gondii* infection alters the brain connectivity microstructure: *in vivo* qualitative and quantitative DT-MRI

**Fig. 2.**
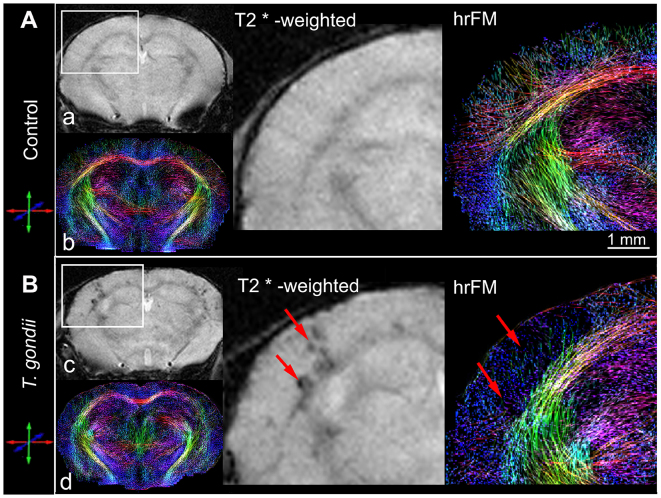
***In vivo* appraisal, by DT-MRI, of cortical injuries induced upon chronic *T. gondii* infection.** Comparative visualization of T2*-weighted images (a,c) and high-resolution fiber maps (hrFM) (b,d) of control (A) and *T. gondii*-infected (B) mouse brains. Magnified views show the localization of *T. gondii*-induced injuries (arrows) in the somatosensory cortex. Note the changed cortical connectivity pattern (versus control) and the loss of fiber density in infected cortical areas. Brain connectivity maps were generated using a global optimization fiber tracking algorithm on data acquired at 9,4 Tesla. (Infected mice *n*=7, control mice *n*=9.)

**Fig. 3.**
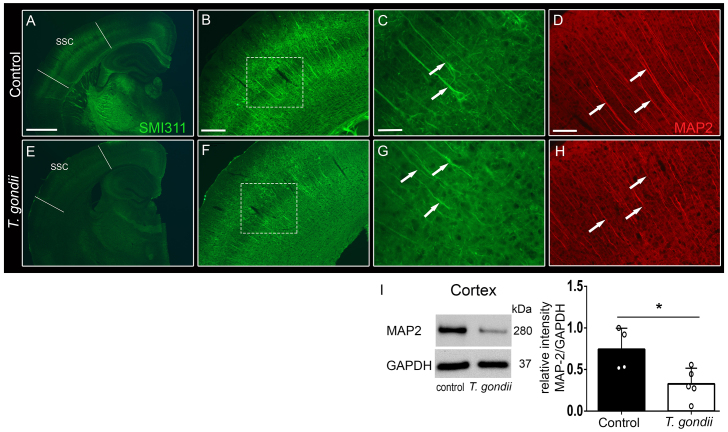
**Structural abnormalities in axons and dendrites were observed within the cortex of *T. gondii*-infected mice.** Immunofluorescence stainings with the neuronal cytoskeleton marker anti-pan-neuronal neurofilament (SMI311) revealed structural abnormalities in the axons and dendritic trees within the SSC (boundaries shown by lines in A,E) of *T. gondii*-infected (E,F) versus control (A,B) mice. Detailed examination of the cortical layers (dashed squares) demonstrated defective morphology of noninfected pyramidal neurons of chronically infected mice (G, arrows), as indicated by reduced expression of SMI311, in contrast to normal expression in control animals (C, arrows). Parallel immunofluorescence staining against microtubule associated protein-2 (MAP2) confirmed the structural alterations of the dendrites within the SSC of *T. gondii*-infected mice (H, arrows) versus control mice (D, arrows). Five to six coronal slides per mouse were analyzed; *n*=3–4 mice per group. Scale bars: 1 mm in A and E, 200 μm in B and F, 50 μm in C and G, 100 μm in D and H. (I) Western blot analysis of MAP2 content in cortical extracts from control and infected mice, alongside GAPDH loading controls. Histograms indicate densitometric analysis of blots, expressed as mean±s.e.m. Analysis was performed in three independent experiments. The circles show individual values, from one representative experiment. *P<0.05.

To investigate the impact of *T. gondii* infection on the mouse brain neuronal wiring, we performed a detailed qualitative and quantitative analysis of brain connectional microstructure using *in vivo* DT-MRI and high-resolution fiber mapping (hrFM). We adopted a novel fiber tracking and mapping methodology (Harsan et al., 2013), which provided fine-grained maps of the living mouse brain structural connectivity (Fig. 2A,B, hrFM). At first, we focused our investigation on the somatosensory cortical areas, showing the highest density of hypointense lesions on T2*-weighted images of the infected mice (Fig. 2). hrFM revealed an altered connectivity pattern with a loss in fiber coherence and density at the lesion sites (Fig. 2B, arrows). Using a special reconstruction algorithm (Reisert et al., 2011; Calamante et al., 2012), the resolution of the final fiber maps was increased by eight times (final resolution of 19.2×19.2×62 μm^3^) versus the scale of the original DT-MRI data. This allows a close comparison with the cortical pattern of axonal cytoskeletal proteins evaluated *ex vivo* with immunofluorescence (Fig. 3). The impaired cortical connectivity blueprints depicted *in vivo* in *T. gondii*-infected brains (Fig. 2B, hrFM) were paralleled by the observation of an abnormal expression pattern of the axonal and dendritic cytoskeleton markers pan-neuronal neurofilament (SMI311; Fig. 3A,F,G) and microtubule associated protein-2 (MAP2; Fig. 3H) in the same cortical areas. Clear reductions of the SMI311 and MAP2 immunofluorescence signals were detected, unraveling structural abnormalities along the cortical (Fig. 3) and hippocampal (supplementary material Fig. S2) dendritic trees in infected mice. Further analysis by western blot revealed a significant reduction in MAP2 content in the cortical extracts in *T. gondii*-infected mice (Fig. 3I; relative intensities normalized to GAPDH: control animals, 0.74±0.12; *T. gondii*-infected mice, 0.32±0.08; *P*=0.02; unpaired Student’s *t*-test). However, such modifications would not only involve the local cortical connectivity, but would influence the overall brain fiber microstructure and wiring.

**Fig. 4.**
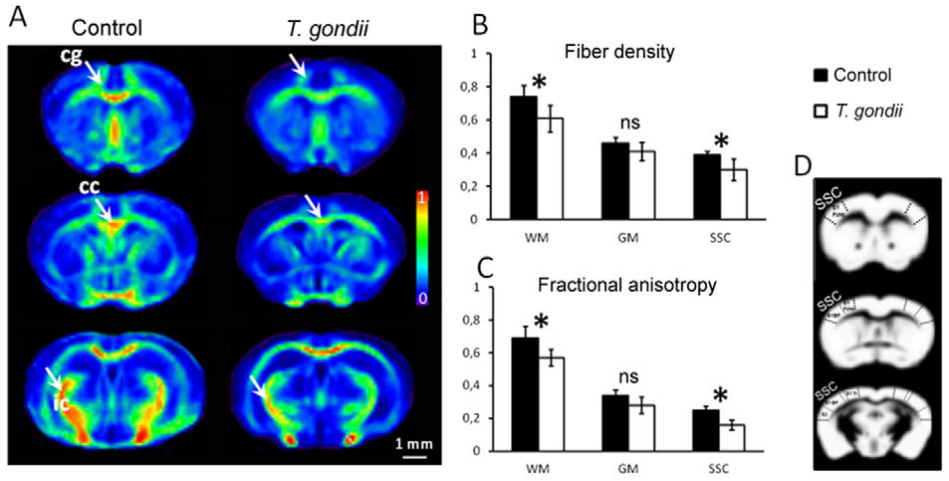
***In vivo* DT-MRI-based quantitative evaluation of brain microstructural alterations induced by chronic *T. gondii* infection.** (A) Group-averaged (control versus *T. gondii*) axial fiber density (FD) maps, generated after spatial normalization to a mouse brain template, show a general pattern of fiber loss particularly evident in white matter areas (arrows; cg, cingulum; cc, corpus callosum; ic, internal capsule) of *T. gondii*-infected mice. The fiber density values were normalized from 0 (no fibers mapped) to 1 (maximum density of fibers depicted in averaged FD maps across the whole population of investigated animals). (B,C) Quantitative comparison of the FD and fractional anisotropy (FA) values in white matter (WM), gray matter (GM) and the somatosensory cortex (SSC) of control and *T. gondii*-infected animals. Note the statistically significant reduction of FD and FA in WM and SSC brain areas upon chronic *T. gondii* infection. Lower FD and FA values were quantified within the GM brain area, without reaching the threshold of statistical significance. (D) Maps illustrate the brain masks [WM (dark area), GM (white area) and SSC] used for the quantitative analysis presented in B and C. The WM and GM masks were generated after segmentation of spatially normalized and averaged T2-weighted images. SSC was manually selected, according to Paxinos mouse brain atlas. All data are expressed as mean±s.e.m.; *T. gondii*-infected mice *n*=7, control mice *n*=9. *P<0.05; ns, not significant.

To obtain quantitative insights about the whole brain structural modifications upon *T. gondii* infection, we performed group statistical analysis (control versus *T. gondii*-infected animals) using the brain parametric maps derived after the calculation of the diffusion tensor as well as the fiber density maps (FD) generated with our global fiber tracking approach. After spatial normalization to a mouse brain template, the brain parametric maps of fractional anisotropy (FA), mean diffusivity (<D>), radial (D┴) and axial (D║) diffusivities and FD from each individual were group averaged and used for quantitative group comparison. Fig. 4 illustrates the overall impact of chronic *T. gondii* infection on the FD and FA values. A general pattern of fiber loss could be noted in the infected group upon comparative inspection of group-averaged FD maps (Fig. 4A). Using a brain-mask approach, we separately assessed the FD modifications in areas of white matter (WM) and gray matter (GM) (Fig. 4C) that were previously segmented from spatially normalized and averaged T2-weighted images. An additional region of interest (ROI) was manually selected, covering the SSC. Statistically significant decreases of the FD values were quantified on the WM area (17.8% reduction: see Fig. 4B; control 0.74±0.07, infected 0.61±0.08; normalized values ranging from 0 to 1) as well as on the SSC (23% reduction: see Fig. 4B; control 0.39±0.03, infected 0.30±0.055; normalized values ranging from 0 to 1). Despite the general tendency of a decrease in FD values over the whole GM brain area, the difference did not reach the statistical significance, mostly because of a high variability across the *T. gondii*-infected mouse population. Comparable patterns of pathologically induced FA alterations (supplementary material Fig. S3A) were quantified along the segmented WM tracts of infected animals (17.6% reduction versus control group: see Fig. 4B; control 0.69±0.07, infected 0.57±0.05; normalized values ranging from 0 to 1) and in the SSC (36% reduction versus control group: see Fig. 4B; control 0.25±0.03, infected 0.16±0.04; normalized values ranging from 0 to 1). Within the entire GM area, no significant FA reduction could be assessed. Interestingly, probing the WM microstructure with electron microscopy revealed myelin abnormalities in the *T. gondii*-infected brains (supplementary material Fig. S3B, left panel). Particularly, we could identify numerous axons displaying clear decompaction and degeneration of the myelin lamellae (supplementary material Fig. S3B, left panel, arrows). Such features, generally considered specific hallmarks of the WM pathology, are illustrated in supplementary material Fig. S3B (arrows) against the normal myelination pattern exemplified in supplementary material Fig. S3B (arrowheads). Moreover, quantification of D┴ values in the WM of infected mice evidenced a statistically significant elevation of the water diffusivity perpendicular to the fiber tracts (supplementary material Fig. S3C; control 0.47±0.08 mm^2^/s, infected 0.58±0.07 mm^2^/s). Increased values of D┴ were previously associated with myelin degeneration (Song et al., 2005; Harsan et al., 2008), suggesting that chronic *T. gondii* infection could elicit pathological processes leading to myelin sheath alterations and, consequently, disrupted structural connectivity. Although subtle modifications of the <D> and D║ were also noticed in the WM, GM and the SSC of *T. gondii*-infected brains (supplementary material Fig. S3C), these parameters do not show enough sensitivity for discriminating the structural modifications induced by the pathology.

### Chronic *T. gondii* infection is negatively influencing the structure of individual, mature cortical and hippocampal neurons

**Fig. 5.**
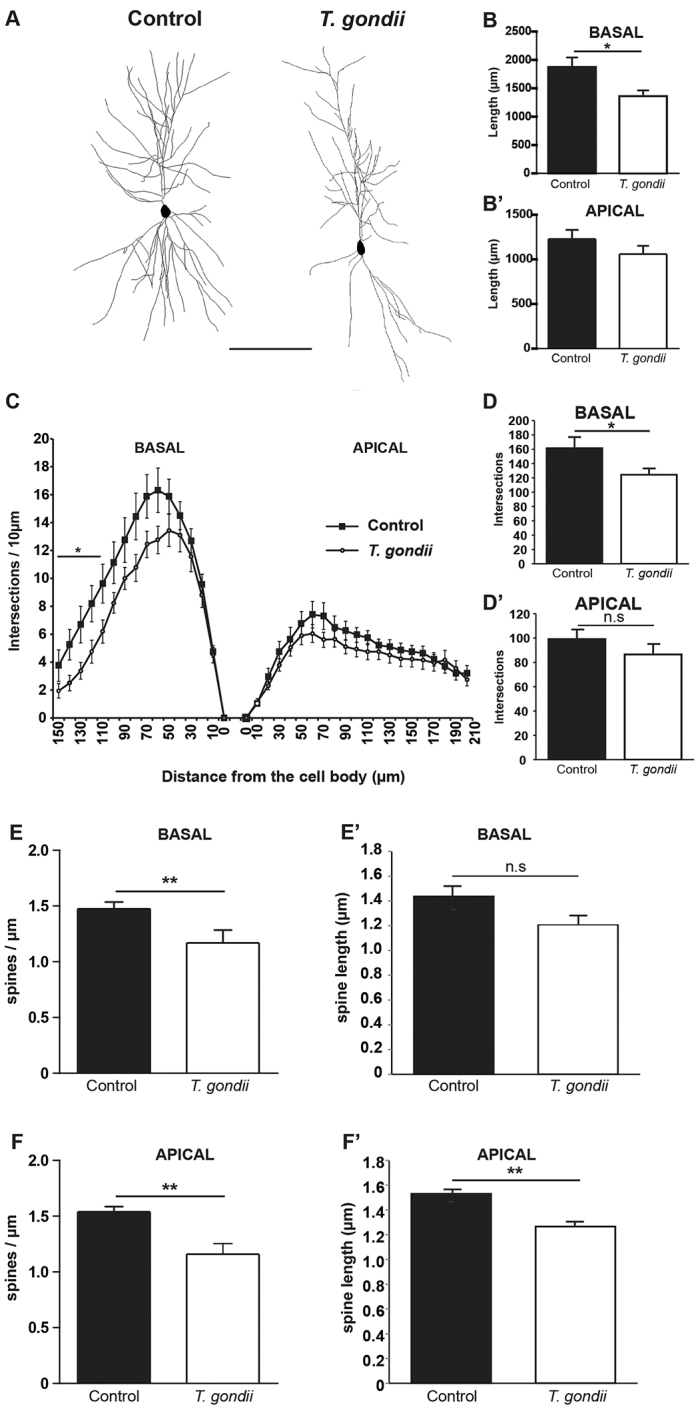
**Morphological analysis of layer II/III pyramidal neurons within the cortices of *T. gondii*-infected and control mice.** (A) Shows two examples of rendered pyramidal neurons, consisting of stacks of multiple optical sections from control and *T. gondii*-infected mice. Note the simplified dendritic architecture in the neuron from the *T. gondii*-infected brain. Scale bar: 100 μm. (B,B′) The graphs compare total dendritic length for the basal and apical dendritic tree in neurons of control versus infected mouse brain. (C) Sholl analysis, plotting dendritic complexity in relation to the distance from the cell body for the basal (left) and apical (right) dendrites of layer II/III neurons of control and infected mouse brain. (D,D′) The graphs show the total dendritic complexity for the basal (D) and apical (D′) dendritic tree of layer II/III neurons of control and infected mouse brain. (E,E′) Depicts dendritic spine density (E) and dendritic spine length (E′) for the basal dendrites of layer II/III neurons of control and infected mouse brain. (F,F′) Show dendritic spine density (F) and dendritic spine length (F′) for the apical dendrites of layer II/III neurons of control and infected mouse brain. Analysis was performed in three independent experiments. All data are expressed as mean±s.e.m.; *n*=3–4 mice per group. *P<0.05; **P<0.01; ns, not significant.

In view of the alterations observed throughout the brains of mice infected with *T. gondii*, we next performed a detailed analysis of the dendritic morphology of individual neurons within two different brain regions. Specifically, we selected layer II/III pyramidal neurons from the cortex and CA1 pyramidal neurons as well as granule cells of the dentate gyrus (DG) from the hippocampus. When qualitatively compared to control layer II/III pyramidal neurons, the cells derived from infected mice showed a very simplified dendritic tree (Fig. 5A). This pattern was confirmed by comparing the total dendritic length (Fig. 5B,B′) and total dendritic complexity (Fig. 5D,D′) using the Sholl analysis. In view of their different morphology and connectivity, we analyzed the complexity of apical and basal dendrites separately. Total dendritic length of layer II/III neurons of *T. gondii*-infected mice was reduced both for the apical and the basal dendrites (Fig. 5B,B′; control apical *n*=20, 1228.36±102.84 μm, infected apical *n*=20, 1059.74±92.42 μm; control basal *n*=16, 1872.45±167.95 μm, infected basal *n*=16, 1357.41±104.17 μm). Moreover, a statistically significant reduction in total dendritic complexity for the basal dendritic compartment could also be observed in infected versus control mice (Fig. 5D,D′; control apical *n*=20, 99.15±8.2 intersections, infected apical *n*=20, 86.5±8 intersections; control basal *n*=16, 161.38±15.11 intersections, infected basal *n*=16, 124.31±8.99 intersections). Finally, a more detailed Sholl analysis performed by plotting the number of intersections against the distance from the cell body confirmed these results (Fig. 5C) and revealed a significant decrease in complexity of the basal dendrites in layer II/III cells of *T. gondii*-infected mice (basal *n*=16; apical *n*=20; unpaired Student’s *t*-test, *P*<0.05, between 100 and 140 μm from the cell body). Next, the density of dendritic spines was analyzed both for the basal (Fig. 5E; control *n*=23, 1.53±0.04 spines/μm, infected *n*=19, 1.15±0.09 spines/μm) and apical (Fig. 5F; control *n*=24, 1.47±0.06 spines/μm, infected *n*=20, 1.16±0.11 spines/μm) dendrites in layer II/III neurons of infected mice and showed a significant reduction when compared with neurons from the same area of control mice. Finally, in order to detect possible changes in the morphology of dendritic spines, their length and head width were compared in pyramidal neurons of control versus infected mice. Although dendritic spine length showed a significant reduction for both the basal and the apical dendrite (Fig. 5E′,F′; control apical *n*=11, 1.53±0.05 μm, infected apical *n*=8, 1.27±0.06 μm; control basal *n*=7, 1.44±0.11 μm, infected basal *n*=5, 1.20±0.06 μm), spine head width was not changed.

Comparing total dendritic complexity of CA1 pyramidal neurons showed a clear reduction upon *T. gondii* infection (supplementary material Fig. S4B,B′; controls *n*=13, infected *n*=15; apical: 25.96%, 95.17±0.8 intersections versus 128.53±11.21 intersections; basal: 29.76%, 99.45±12.97 intersections versus 141.58±14.53 intersections). Total dendritic complexity was also reduced in granule cells of the DG (supplementary material Fig. S4D; 17.42%, 84.81±4.39 intersections versus 70.03±4.36 intersections; *P*<0.05). Moreover, whereas total dendritic length was only mildly reduced in CA1 neurons of *T. gondii*-infected mice compared with controls, it was significantly lower in granule cells. Detailed Sholl analysis, performed in the DG of infected mice, showed a clear reduction in dendritic complexity (supplementary material Fig. S4C) throughout the entire dendritic length (160 μm from the cell body). Similarly, the Sholl analysis revealed a decrease in complexity of the apical and basal dendrites of CA1 pyramidal neurons of infected versus control mice, throughout the analyzed length (supplementary material Fig. S4A).

### Synaptophysin and PSD95 protein expression is reduced in the cortex and hippocampus of mice chronically infected with *T. gondii*

**Fig. 6.**
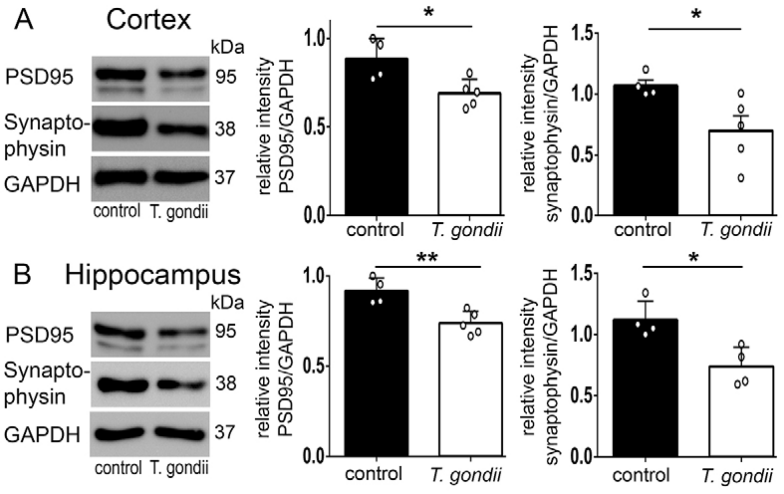
**Impaired expression of synaptic-related proteins in *T. gondii*-infected brains.** Representative blots from the cortex (A) and hippocampus (B) tissue extracts of control and *T. gondii*-infected mice, used for PSD95 (thick band) and synaptophysin protein semi-quantification. Densitometric analysis of PSD95 and synaptophysin protein levels revealed statistically significant reductions in *T. gondii*-infected mice, suggesting deficits of the synaptic cleft physiology. Analysis was performed in three independent experiments. Data are expressed as mean±s.e.m. The circles show individual values from one representative experiment. *P<0.05 and ** *P*<0.01.

To determine whether the observed changes in neuronal architecture might be reflected in altered expression of key molecules involved in synaptic signaling, we investigated the expression of presynaptic synaptophysin as well as the postsynaptic density protein (PSD95) by western immunoblotting. Semi-quantitative protein expression revealed significantly lower protein levels of these two molecules between *T. gondii*-infected and noninfected animals, in both cortex and hippocampus (Fig. 6A,B) (relative intensities normalized to GAPDH: PSD95, cortex: control animals, 0.88±0.05; *T. gondii*-infected mice, 0.69±0.03; *P*=0.01; PSD95, hippocampus: control animals, 0.91±0.03; *T. gondii*-infected mice, 0.73±0.02; *P*=0.005; synaptophysin, cortex: control animals, 1.06±0.04; *T. gondii*-infected mice, 0.69±0.12; *P*=0.03; synaptophysin, hippocampus: control animals, 1.11±0.07; *T. gondii*-infected mice, 0.73±0.07; *P*=0.01; unpaired Student’s *t*-test). Our findings provide evidence that chronic *T. gondii* infection can interfere with the normal activity of mature synapses in key brain structures.

## DISCUSSION

Behavioral changes triggered by *T. gondii* in rodents have been extensively discussed in recent years (Hutchinson et al., 1980; Webster et al., 1994; Berdoy et al., 1995; Berdoy et al., 2000; Vyas et al., 2007; McConkey et al., 2013; Worth et al., 2013). Several clinical studies claimed a possible association between *T. gondii* seropositivity and neurological diseases in humans, but no sufficient explanation or underlying mechanisms has been described to date (Flegr et al., 2002; Torrey and Yolken, 2003; Brown, 2006; Wang et al., 2006; Yereli et al., 2006; Hinze-Selch et al., 2007; Arling et al., 2009; Zhu, 2009; Hinze-Selch et al., 2010; Pedersen et al., 2012; Fabiani et al., 2013). These specific behavioral and neurological changes occur, not exclusively, owing to focal brain abnormalities associated with parasite cyst localization, but might be a result of pathological interactions between different brain areas. Furthermore, the dysregulation of the inflammatory milieu, neurotransmitter imbalance, as well as synaptic plasticity could contribute to this effect. Converging evidence provided by advances in imaging techniques in recent years confirms a close relationship between disturbances in brain networks and debilitating neuropsychiatric disorders in humans with cognitive and psychomotor impairments (Lawrie et al., 2003; Goghari et al., 2005; Menzies et al., 2008; Vasic et al., 2009; Zalesky et al., 2011; Woodward et al., 2012; Anticevic et al., 2013). The core pathology of dysconnectivity syndromes resides mostly in aberrant synaptic plasticity due to disturbed interaction between neurotransmitters and different immunological factors (Coogan and O’Connor, 1997; Curran and O’Connor, 2001; Stephan et al., 2006). Therefore, we have explored the impact of *T. gondii*-induced microlesions on the whole mouse brain neuronal circuitry as well as on the morphology of individual noninfected neurons, and we correlated the neuroanatomical alterations in those areas with changes in the synapse efficacy as possible contributing factors for the behavioral and neurological deficits.

In contrast to other studies, which have investigated the location of *T. gondii* cysts in specific brain areas, we focused our analysis on the position of *T. gondii*-induced lesions. These lesions are detectable in a variety of neuroanatomical areas where complex neuro-immunological dynamics occur. Initially, we combined the conventional MRI (based on T2- and T2*-contrasts) and DT-MRI with a global fiber tracking algorithm in order to identify the brain areas with the highest density of *T. gondii*-induced lesions and to further assess their impact on the neuronal circuitry pattern. The SSC, motor cortices, hippocampus and striatal brain structures were depicted as primary sites with altered T2*-MR based contrast, suggesting substantial neuroanatomical changes within these brain regions of the infected mice. A previous study by Hermes et al. (Hermes et al., 2008) evaluated the chronic *T. gondii* infection by T2-weighted MRI; however, they observed only moderate ventricular dilatation in infected mice. The reason for the disparate findings could be that T2-based MR contrast is less sensitive compared to T2*-weighted MRI for visualizing the brain microlesions. Alternatively, the genetic resistance of Swiss Webster mice could also play an important role.

Histological examination revealed that *T. gondii*-infection-induced lesions were constituted by activated resident glia cells, as well as recruited mononuclear cells and a few neutrophil granulocytes and lymphocytes, similar to the description by Gulinello et al. (Gulinello et al., 2010). Additionally, only a small number of neurons in the affected areas were degenerated, suggesting that the majority of them remained viable. We observed random distribution of *T. gondii* cysts, with no preference to specific brain areas, corresponding with the previous results by Gulinello et al. (Gulinello et al., 2010) and Berenreiterová et al. (Berenreiterová et al., 2011), whereas other groups reported elevated cyst numbers in certain brain regions of infected animals (Vyas et al., 2007; Haroon et al., 2012). Furthermore, the location of the cysts was also random in one recent study investigating a rat model in which the infection is associated with limited inflammation (Evans et al., 2013). In our experiments, the low number of *T. gondii* cysts and the corresponding moderate number of infected neurons suggest that, in addition to the infected cells, other components might alter neuronal morphology and function. It is well described that the encysted intracellular parasites indirectly influence the release of soluble mediators such as cytokines, chemokines, growth factors and brain-specific proteins (Strack et al., 2002; Suzuki, 2002a; Hunter and Sibley, 2012). These factors, secreted by resident glial cells as well as recruited immune cells, could specifically influence neuronal function and communication. It was previously described that increases of pro-inflammatory cytokines lead to reduced synaptic plasticity (Coogan and O’Connor, 1997; Curran and O’Connor, 2001). In the case of chronic *T. gondii* infection, it is possible that continuous production of inflammatory molecules will lead to changes in synaptic plasticity and abnormal wiring of brain connections. Further detailed studies should identify the specific pathophysiological mechanisms as well as the exact character and source of inflammatory mediators.

Detailed qualitative and quantitative analysis of neuronal fibers using DT-MRI and hrFM revealed the impact of *T. gondii* infection on regional and global mouse brain connectional microstructure. This novel approach is currently the only methodology providing a noninvasive window into the whole brain structural connectivity (Harsan et al., 2013). We took advantage of this new technique and demonstrated not only a loss of fiber continuity and density at lesion sites, but also substantial alterations in the general wiring scheme. This involved GM cortical and subcortical changes as assessed by the reduced FD and FA values, but also significant abnormalities along major WM tracts. DT-MRI findings in SSC were paralleled by modifications of neuronal cytoskeletal protein levels, verifying the validity of our *in vivo* approach. Notably, infected mice displayed reduced expression of neurofilament in the SSC and hippocampus. Qualitative evaluation of immunofluorescence images revealed a clear reduction of SMI311 and MAP2 immunoreactivity, confirming abnormalities along the cortical and hippocampal dendritic trees. Importantly, quantification of MAP2 content in the cortex by western blot confirmed the reduced expression levels of this protein. Given the roles of neurofilament and microtubules in the radial growth of axons and dendrites, leading to the stabilization and maturation of neuronal connections (Sánchez et al., 2000; Al-Chalabi and Miller, 2003; Penzes et al., 2009), we suggest that the altered expression of these two proteins supports the existence of abnormalities in axonal and dendritic morphology in the SSC with possible functional consequences on behavior.

Moreover, our analysis unveiled a pattern of *T. gondii*-induced white matter pathology. We showed such modifications, first by *in vivo* DT-MRI and then by electron microscopy. Reduced FA values in white matter areas along with significantly increased radial diffusivity were associated with myelin abnormalities. The electron micrographs revealed clear decompaction as well as degeneration of the myelin lamellae in *T. gondii*-infected brains. To our knowledge, evidence of *T. gondii*-induced white matter microstructural changes has not been shown previously. Interestingly, white matter changes revealed by DT-MRI were located along important tracts, such as the corpus callosum, cingulum and internal capsule. Impaired inter-hemispherical callosal connectivity would certainly functionally affect the somatosensory system, given the direct connections of the left and right SSC through this pathway. Cingulum is another fiber pathway known for allowing communication between components of the limbic system and projecting in areas such as the entorhinal cortex or hippocampus. Such pathological processes characterized by white matter structural alterations in the mentioned brain areas are likely to contribute to functional impairments associated with behavioral modifications.

To further explore the structural alterations underlying the changes in the brain connectivity, we focused our study to closer investigate the morphological modifications suffered by noninfected pyramidal neurons in the somatosensory cortical layers II/III, upon chronic infection with *T. gondii*. Pyramidal cells in layer III are excitatory neurons involved in projections between various brain areas contributing to fundamental sensory and cognitive processes. Therefore, precise morphological analysis of individual pyramidal neurons from the indicated areas revealed impaired dendritic branching both for the apical and the basal dendrites in infected mice compared with noninfected controls. Extending these findings to the hippocampus, we report a reduction of total dendritic complexity of the neurons located in the CA1 and DG regions, suggesting that these changes might be intimately related to dysfunctions in the memory and learning processes of mice chronically infected with *T. gondii*. Changes in dendritic morphology predict changes in the number of dendritic spines that are responsible for information exchange. Dendritic spines are specialized membrane protrusions located on neuronal dendrites; they have a very complex structure and fundamental functions in the synaptic physiology and plasticity (Nimchinsky et al., 2002). Spine abnormalities have been observed in several neurological and psychiatric disorders (Glantz and Lewis, 2000; Nimchinsky et al., 2002). We found a significant reduction in the number of spines within the pyramidal neurons of layers II/III for apical, as well as for basal, dendrites. Morphological changes such as significant reduction in spine length was detected. Because layer II/III pyramidal cells are intercortical neurons, the loss of dendritic spine number and morphological abnormalities can be associated with disrupted communications between higher cortical areas, thus providing a possible explanation for the behavioral alterations. Our results suggest that neuronal changes in chronic *T. gondii* infection is a consequence of the interplay between the host immune system and the CNS, rather than a direct effect of the parasites on individual neurons within a specific brain region. Additional methods will need to be employed to test the functional implications of these findings and to clarify the precise nature of those alterations in *T. gondii*-infected mice.

Importantly, we measured significantly lower synaptophysin and PSD95 protein levels in infected mice compared with noninfected mice by western blot, indicating clear alteration in integrity and functionality within mature synapses of mice infected with *T. gondii*. Synaptophysin is involved in modulation of the efficiency of the synaptic vesicle cycle (Valtorta et al., 2004), whereas the function of PSD95 is the correct assembly of the postsynaptic density complex (Chetkovich et al., 2002; Cao et al., 2005). A recent study using synaptophysin-deficient mice reported impaired memory and spatial-learning capacities and reduced novelty recognition, similar to mice infected with *T. gondii* (Schmitt et al., 2009). Decreased expression of synaptophysin associated with cognitive decline has been described in the adult rat brain in a model of chronic induced neuroinflammation (Deng et al., 2012). PSD95 activity is tightly linked to normal regulation of synaptic plasticity (Montgomery and Madison, 2004). Abnormalities in PSD95 and related postsynaptic-density-complex molecules can contribute to abnormal synaptic plasticity, which structurally correlates to changes in the morphology or distribution of dendrites and dendritic spines (Rajan and Cline, 1998; Monfils and Teskey, 2004). Overall, synaptic damage and functional loss can lead to altered information trafficking between the structures of the CNS and might contribute to the reported behavior and neuropsychiatric changes observed in chronic *T. gondii* infection.

Our analysis has revealed for the first time evidence of defined modifications in the brain microstructure and neuroconnectivity, in co-occurrence with synaptic efficacy alterations, in a murine model of chronic *T. gondii* infection. Given the specificity of our results, we suggest an important role for synaptic plasticity in the behavioral changes and neuropsychiatric disorders associated with chronic *T. gondii* infection.

## MATERIALS AND METHODS

### Animals

All animal experiments were approved, according to German and European legislation by the Landesverwaltungsamt Halle (Sachsen-Anhalt, Germany, approval number IMMB/G/01-1089/11). Experiments were conducted on adult C57BL/6 female mice (8 weeks old, purchased from Janvier).

### T. gondii infection

*T. gondii* cysts of the ME49 type II strain were used for this study. Parasites were harvested from the brains of NMRI mice infected intraperitoneally (i.p.) with *T. gondii* cysts 4 to 5 months earlier. Brains obtained from infected mice were mechanically homogenized in sterile phosphate-buffered saline (PBS) and the cysts counted using a light microscope. Three cysts were administered i.p. into mice in a total volume of 200 μl/mouse. Control mice were mock infected with sterile PBS.

### Experimental design

Mouse brain T2- and T2*-weighted images were acquired at different time points. The development and localization of *T. gondii*-induced brain lesions were monitored starting 1 week prior to infection (week −1) to chronic stages [week 8 post-infection (p.i.)] and validated by histopathological examination.

At the chronic stage (week 8 p.i.), *in vivo* mouse brain DT-MRI analyses were additionally performed in *T. gondii*-infected and control (noninfected) mice. At the same timepoint, three to four subjects per group, in up to three independent experiments, were used for immunohistopathological investigation, western blotting, individual neuron morphology analysis and electron microscopy.

### MRI data acquisition and post-processing

Mouse brain MRI was performed on seven infected mice (week 8 p.i.) and nine control mice using a 9.4 T small bore animal scanner, equipped with a BGA12S gradient system capable of 675 mT/m (BioSpec 94/20, Bruker, Germany). A transmit/receive 1H mouse quadrature birdcage resonator (35 mm inner diameter) and the ParaVision 5 software interface were also used for the acquisition of the MR signal. All imaging procedures were carried out under isoflurane (3% for induction and ~1.5% for maintenance) mixed with oxygen (1 l/min) anesthesia and using respiratory gating.

### Morphological T2*- and T2-weighted imaging

A FLASH 2D sequence was used for T2*-weighted imaging of the whole mouse brain in 31 contiguous axial slices, acquired with a 78×78×500 μm^3^ imaging resolution (TR/TE=920/12 ms, flip angle=60°, 2 signal averages). Acquisition time: 7m52s. RARE T2-weighted imaging was performed with the same slice geometry and resolution as described for T2*-imaging, using the following parameters: TR/TE=3675/20 ms, RARE factor=4 and 4 signal averages, acquisition time: 15m40s.

### DT-MRI

Diffusion MRI data was acquired in 31 axial 500-μm slices, with the same orientation as for the previous T2*- and T2-weighted scans, using a 4-shot DTEPI sequence [repetition time, 7750 ms; echo time, 20 ms; time (∆) between the application of diffusion gradient pulses, 14 ms; diffusion gradient duration (δ), 7 ms]. The acquisition protocol was adapted for using diffusion-sensitizing gradients applied along 45 isotropic directions of three-dimensional space, with a b factor of 1000 seconds/mm^2^. Six averages were acquired to increase the signal-to-noise ratio (SNR). The in-plane image resolution was 156×156 μm at a field of view (FOV) of 20×20 mm and an acquisition matrix of 128×92. The resulting total imaging time for the T2*-weighted, T2-weighted and DT-MRI was ~2 hours 20 minutes for each animal.

DT-MRI data post-processing, including tensor calculation, generation of parametric maps (fractional anisotropy, FA; mean diffusivity, <D>; radial, D┴, and axial, D║, diffusivities) and fiber tracking was performed using a FiberTool package developed in-house (http://www.uniklinik-freiburg.de/mr/live/arbeitsgruppen/diffusion_en.html).

A global fiber tracking approach was employed to define the large-scale mouse brain connectivity networks (Harsan et al., 2013). The global tracking algorithm reconstructed all fiber bundles simultaneously, for the whole brain, without the requirement of defining seed or target regions (Reisert et al., 2011). The results were used for generating mouse brain fiber density maps (FD) as well as highly resolved spatial histograms of diffusion orientations – here named high-resolution fiber map (hrFM). hrFM were tailored to increase eight times the native imaging resolution of the diffusion data, reaching a spatial resolution of 19.5×19.5×62.5 μm^3^. We used a reconstruction approach previously described by us and another group (Calamante et al., 2012; Harsan et al., 2013).

### Quantitative comparison of *T. gondii* versus control group

Spatial normalization of the mouse brain parametric maps generated from the diffusion data (including FA and FD maps) and the morphological T2*-and T2-weighted images was performed using Matlab-script, which registered the brain maps from all investigated animals on a previously created mouse brain template. Group (*T. gondii* versus control group)-averaged maps of FA, FD, <D>, D┴ and D║ were created and used for quantitative group comparison. White matter (WM), gray matter (GM) and cerebrospinal fluid (CSF) masks were generated using the T2-weighted images and used as ROI. An additional ROI, the somatosensory cortex (SSC), was included in the quantification and was manually selected based on Paxinos atlas (Paxinos and Franklin, 2001).

### Brain harvesting

Mice were anesthetized by i.p. injection of ketamine-xylazine (100 mg/kg ketamine, 5 mg/kg xylazine) and perfused intracardially with sterile PBS. In addition, brains were isolated from infected mice and dispersed in 1 ml sterile PBS by mechanically disrupting the tissue. Finally, cysts were counted microscopically in 10 μl of the brain suspension and the total number of cysts per brain was calculated.

### Histopathological analysis

Brains were immediately removed from the skull, embedded in paraffin and sectioned coronally at 5 μm. To evaluate the lesions observed by MRI, a classical examination was performed with hematoxylin and eosin (H&E). Primary antibodies against Iba1 (1:250, Dako, Denmark), CD3 (1:150, Dako, Denmark), CD11b (1:200, Abcam, UK), GFAP (1:200, Abcam, UK), cleaved caspase-3 (Asp175, 1:200, Cell Signaling, USA) and *T. gondii* (1:200, Dianova, Germany) were used to assess inflammation, astrogliosis and neurodegeneration.

### Immunofluorescence

For immunofluorescence and Fluoro-Jade B, brains were sectioned coronally (18 μm) using a Leica cryostat (C 3050, Wetzlar, Germany). Immunolabeling with a mouse anti-pan-neuronal neurofilament (1:800, SMI311, Millipore, Calbiochem, USA) and rabbit anti-microtubule-associated protein (1:50, MAP2, Cell Signaling, USA) antibodies were performed overnight at 4°C.

Tissue sections were incubated with appropriate (anti-rabbit/anti-mouse) Alexa-Fluor-488- or 594-conjugated secondary antibody (1:200, Invitrogen, Germany) and mounted with ProLong Gold (Invitrogen, Germany). A Zeiss (Carl Zeiss, Germany) microscope equipped with an AxioCam HRc 3 digital camera and AxioVision 4 Software were used to obtain images. A minimum of six region-matched slices were analyzed per brain. Regions were assigned according to a mouse stereotaxic atlas (Paxinos and Franklin, 2001).

### FluoroJadeB staining

Fluoro-Jade B (Millipore, Chemicon, USA) staining was performed according to the manufacturer’s indications with slight modifications. Sections were immersed in 100% ethanol for 5 minutes followed by 70% ethanol (2 minutes) and distilled water (2 minutes). The rehydration step was followed by incubation in 0.06% potassium permanganate (15 minutes), then rinsed in dH2O and transferred for 30 minutes in 0.0004% Fluoro-Jade B solution in the dark. Finally, the sections were rinsed three times in distilled water and cleared in Roti-Histol (Roth, Germany) before coverslipping with DPX (Fluka, Milwaukee, WI). On average, four region-matched sections were analyzed per brain.

### Electron microscopy

Coronal brain slides (150–200 μm) corresponding to the mid third of the cerebrum were cut immediately after perfusion and fixed in phosphate-buffered glutaraldehyde, followed by embedding in epon. Toluidine-blue-stained sections (500 nm thickness) were evaluated for the optimum area for electron microscopic examination. Ultrathin sections (50 nm) were cut using a Leica Ultracut device, mounted on copper grids and stained with lead citrate solution for contrast enhancement. Sections were evaluated on a Zeiss-EM 900 electron microscope.

### DiOlistics and morphological analysis

Cortical and hippocampal neurons from control and chronically infected mice were labeled using DiOlistic on fixed acute slices (Rauskolb et al., 2010; Heneka et al., 2013). After anesthesia, animals were perfused with 4% PFA and 4% sucrose in PBS and the brains were dissected and post-fixed in the same solution for 30 minutes at 4°C. Next, the brains were embedded into 2% agar in PBS and cut into 400-μm-thick coronal sections with a vibratome (Leica VT 1000 S). Tungsten particles (50 mg; 1.7 μm in diameter; Bio-Rad) were mixed with dye solution (3 mg DiI, Invitrogen, in 100 μl of methylene-chloride, Sigma-Aldrich), dried and suspended in 3 ml distilled water. The dye solution was sonicated, diluted 1:80 and used to coat the inside of a TEZFEL tubing (Bio-Rad, Germany). Dye-coated particles were delivered to the acute slices using a hand-held gene gun (Bio-Rad; Helios Gene Gun System) with a pressure of 120 psi. After shooting, the slices were kept in PBS for 6 hours at room temperature (RT) to allow dye diffusion. The neurons were imaged with an Axioplan 2 imaging microscope (Zeiss, Germany) equipped with an ApoTome module (Zeiss, Germany). Morphological reconstruction of the neurons and their processes were achieved with the Neurolucida software (MicroBrightField). Dendritic complexity and length of the dendrites were analyzed with the Neuroexplorer software (MicroBrightField). The cells were analyzed up to a distance of 160 μm for DG, 200 μm for CA1 apical and cortex layer II/III apical and 150 μm for CA1 basal and cortex layer II/III basal, to ensure that only completely labeled dendrites were included in the analysis. The data obtained were compared using a two-tailed Student’s *t*-test.

### Western blot analysis

For each individual brain, the hippocampus and the ipsilateral cortex were dissected as described previously (Stoppini et al., 1991; Gogolla et al., 2006). Protein content was determined using the Bicinchoninic Acid protein assay kit (Pierce, Rockford).

Samples containing equal amounts of total protein (10.0 μg) were separated on 10% SDS-polyacrylamide gels and transferred to polyvinylidene fluoride membranes. For immunoblotting, the following primary antibodies were used: monoclonal rabbit anti-PSD-95 (1:1000; Cell Signaling, USA), monoclonal rabbit anti-synaptophysin (1:5000; Cell Signaling, USA), monoclonal rabbit anti-MAP2 (1:1000; Cell Signaling, USA) and monoclonal rabbit anti-GAPDH (1:10,000; Cell Signaling, USA). Secondary antibody was horseradish peroxidase (HRP)-linked polyclonal swine anti-rabbit (1:1700; Dako, Denmark). Blots were developed using a Pierce ECL 2 kit (Thermo Scientific, USA).

For semi-quantitative studies, the chemiluminescence signals were captured using a ChemoCam Imager (Intas, Germany). The immunostained band densities were normalized with respect to the loading control (GAPDH) and analyzed using LabImage software (Kapelan Bio-Imaging, Germany). Data were analyzed with GraphPad Prism 6. Statistical significance was determined using the unpaired two-tailed Student’s *t*-test.

## Supplementary Material

Supplementary Material
