# Supplementary material for: Chronic murine toxoplasmosis is defined by subtle changes in neuronal connectivity
Source: Dis Model Mech. 2014 Feb 13;7(4):459–69. doi: 10.1242/dmm.014183 (PMC3974456; doi:10.1242/dmm.014183)
Supplement: Supplementary Material [file supp_7_4_459__index.html]

Chronic murine toxoplasmosis is defined by subtle changes in neuronal connectivity — Supplementary Material 

# Chronic murine toxoplasmosis is defined by subtle changes in neuronal connectivity

## DMM014183 Supplementary Material

**Files in this Data Supplement:**

- **Supplementary Material**
