# Supplementary material for: Chronic murine toxoplasmosis is defined by subtle changes in neuronal connectivity
Source: Dis Model Mech. 2014 Feb 13;7(4):459–69. doi: 10.1242/dmm.014183 (PMC3974456; doi:10.1242/dmm.014183)
Supplement: Supplementary Material [file supp_7.4.459_DMM014183.pdf]

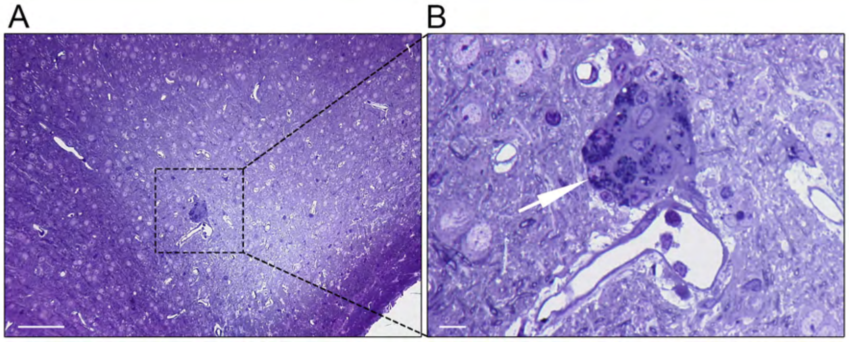

Fig. S1 Toluidine blue staining from a T. gondii infected mouse cortex, depicting a macrophage scavenging damaged myelin. Magnified view shows the perivascular macrophage (arrow) containing numerous phagosomes filled with residues of myelin sheats. (Scale bars, 100 μm in A and 10 μm in B)

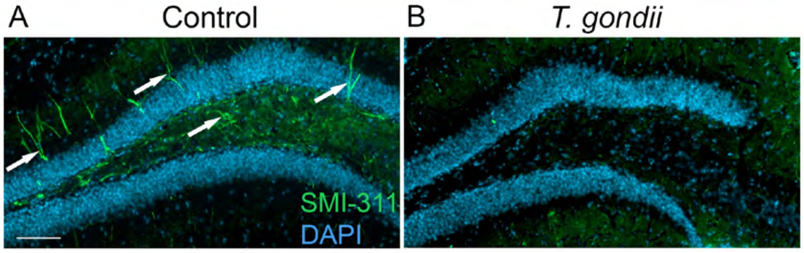

Fig. S2 Chronic T. gondii infection strongly reduces the expression of neurofilament within the hippocampus of infected mice. (A) Anatomically normal dendrites (arrows) are found in the dentate gyrus of control mouse hippocampus slides stained with anti-Pan-Neuronal-Neurofilament (SMI311) marker. (B) Severe structural anomaly in the dendritic arborization of the dentate gyrus could be assessed histologically in T. gondii infected brains. Analysis was performed in 3 independent experiments, n= 3-4 mice per group; five to six coronal slides per mouse were analyzed. (Scale bar, 100 μm)

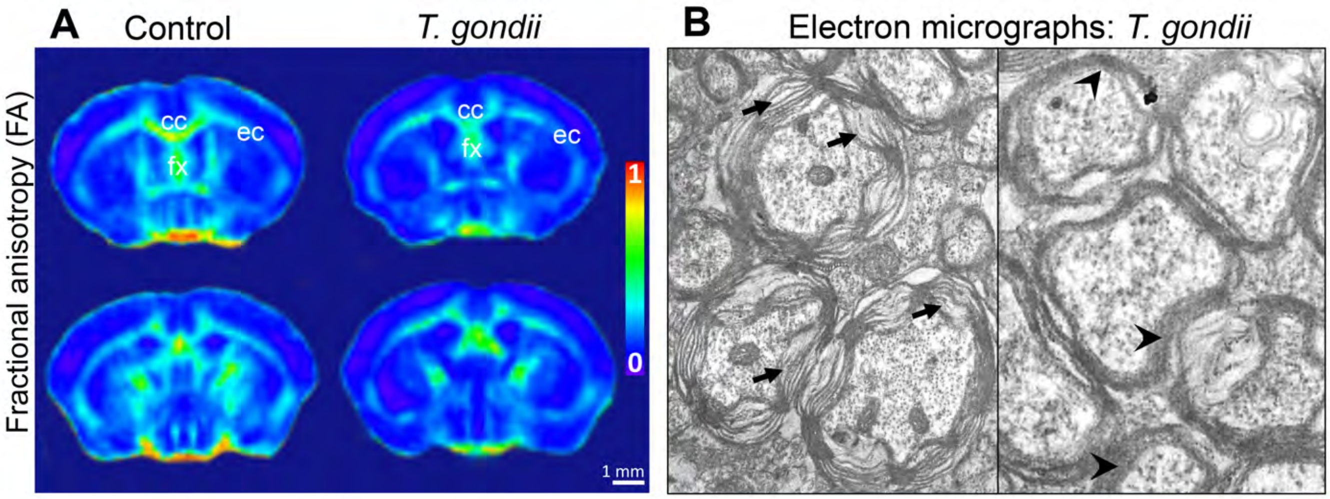

C

ROIs	WM		GM		SSC	
Groups	Control	*T. gondii*	Control	*T. gondii*	Control	*T. gondii*
<D> (x10^{-3} mm^2/s)	0.87±0.11	0.97±0.10	0.70±0.07	0.73±0.1	0.65±0.05	0.71±0.08
	ns		ns		ns	
$D_{\perp}$ (x10^{-3} mm^2/s)	0.47±0.08	0.58±0.07	0.65±0.07	0.69±0.1	0.60±0.05	0.66±0.08
	*		ns		ns	
$D_{\parallel}$ (x10^{-3} mm^2/s)	1.31±0.14	1.40±0.12	0.75±0.08	0.77±0.07	0.70±0.05	0.75±0.07
	ns		ns		ns	

Fig. S3 Quantitative assessment of modifications induced by T. gondii infection on brain parametric maps generated from in vivo DT-MRI data. (A) Comparison of group averaged axial FA maps (control vs. T. gondii infected groups) within two different brain locations, showing decreased FA values along the WM tracts (i.e. cc – corpus callosum; ec – external capsule; fx – fornix). (B) Electron micrographs from T. gondii infected brain, depicting axons with myelin lamellae decompaction (left panel – arrows). Intact myelin sheath surrounding healthy axons are comparatively shown in the right panel – arrowheads. (C) Detailed quantitative comparison of mean (<D>), radial ($D\perp$) and axial ($D\|$) diffusivities in white matter (WM), gray matter (GM) and somatosensory cortex (SSC) of control and T. gondii infected animals. $D\perp$ is the only parameter showing statistically significant increase in the WM of infected brains. Note also the general trend of elevated mean diffusivity <D> in all investigated regions of interest (ROIs). All data are expressed as means ± SEM, T. gondii infected mice n= 7, control mice n= 9. *p<0.05; ns, not significant.

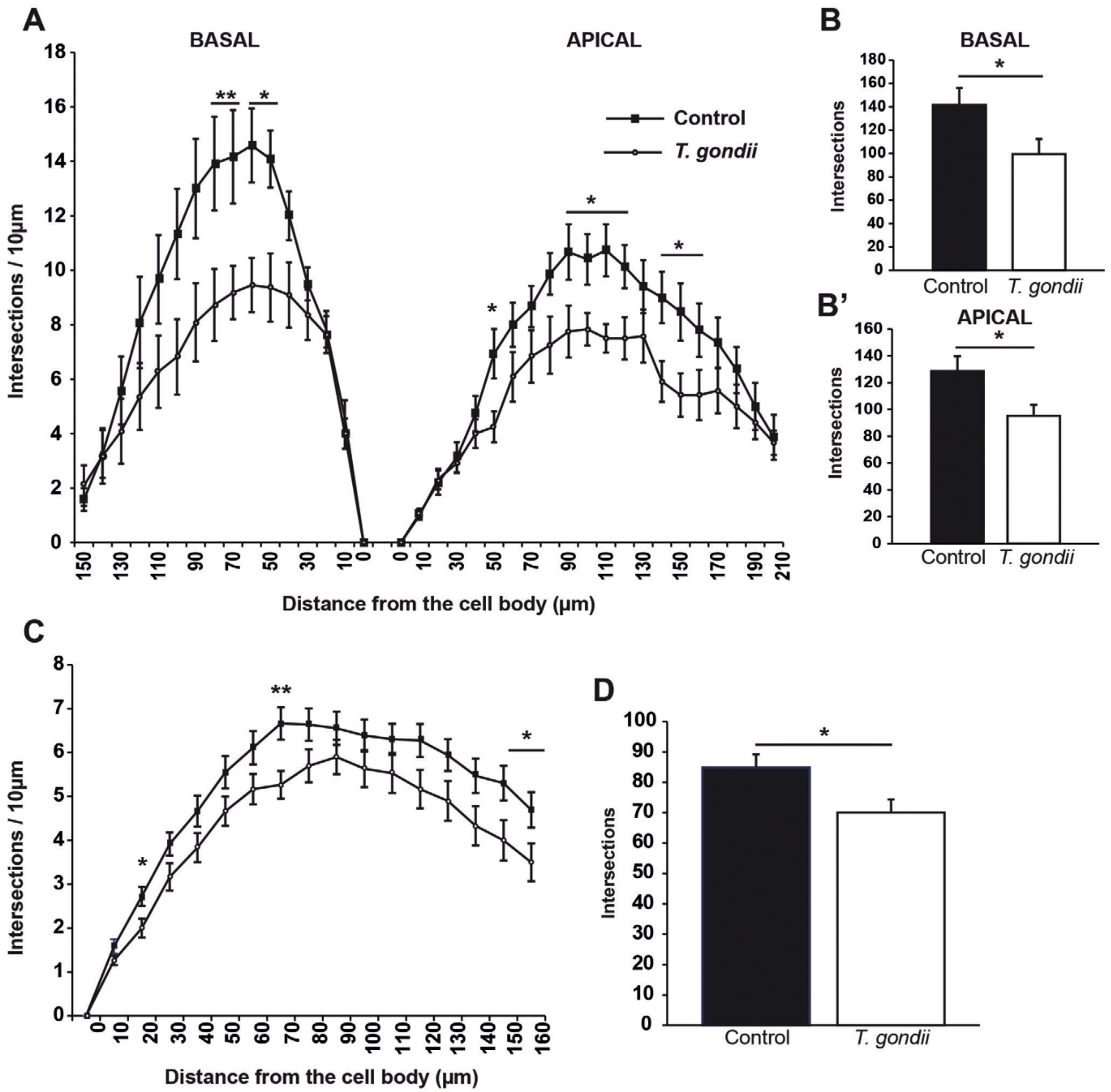

Fig. S4 Morphological analysis of neurons within the hippocampus of T. gondii infected and control mice. (A) Sholl analysis, plotting the distribution of dendritic complexity in relation to the distance from the cell body for the basal (left) and apical (right) dendrites of CA1 pyramidal neurons of control and T. gondii infected brain. (B), (B') the graphs show the total dendritic complexity for the basal (B) and apical (B') dendritic tree of CA1 pyramidal neurons of control and T. gondii infected brain. (C) Sholl analysis, plotting the distribution of dendritic complexity in relation to the distance from the cell body of neurons from the dentate gyrus of control and T. gondii infected mice. (D) The graph shows the total dendritic complexity for the dendritic tree of granule neurons in the dentate gyrus of control and infected brains. Analysis was performed in three independent experiments. All data are expressed as means ± SEM, n= 3-4 mice per group.*p<0.05; **p<0.01.
